# Kawasaki disease epidemic: pitfalls

**DOI:** 10.1186/s13052-020-00887-4

**Published:** 2020-08-27

**Authors:** Romina Gallizzi, Giovanni Corsello, Giovanni Battista Pajno

**Affiliations:** 1 Department of Human Pathology of Adulthood and Childhood Gaetano Barresi, Gaetano Martino University Hospital, UOC Pediatria, University of Messina, Via Consolare Valeria, 98125 Messina, Italy; 2 Department of Maternal and Child Health, University of Palermo, Palermo, Italy

**Keywords:** Kawasaki disease, SARS-CoV-2, Hyperinflammatory response, Pediatric population

## Abstract

Recent reports have described in the pediatric population a new type of hyperinflammatory response manifested following contact with SARS-CoV-2, with some of the clinical features attributable to Kawasaki disease (KD). The purpose of this commentary is to remark on a possible recent association between SARS-CoV-2 and KD. Although today little is known about the etiology of KD, the most accepted hypothesis is that of a probable viral etiology, therefore, even the SARS-CoV-2 virus could trigger, in genetically predisposed subjects, an exaggerated inflammatory response that is clinically evident like the one described in KD.

## Background

Kawasaki disease (KD) is a vasculitis of the small and medium caliber vessels with a preference for coronary arteries and it is the most common cause of heart disease acquired in children [1]. The diagnosis of KD remains clinical and there are no specific laboratory tests; the American Heart Association (AHA) criteria and guidelines reviewed in 2017 are used [2]. The cause of KD remains unknown. A careful study connects the seasonality of KD to tropospheric wind patterns, that provides the transportation of an agent which, if inhaled by genetically sensitive children, it triggers the KD immunological cascade. Another study suggests new RNA virus infection that enters the upper respiratory tract [2]. Activation of the innate immune system is an initial event, with evidence of the activation of the interleukin signal pathway (IL-1, IL-6 and TNF-alpha) [3]. The self-limited nature of the disease combined with a low rate of recurrence suggests the rotation of T and B memory cells which are protective against future encounters with the agent of KD [4]. KD has been reported from more than 60 countries across the world, around the equator to areas near the poles and is seen in both hot and cold areas. This confirms that several infectious agents may trigger the disease in different geographical areas and seasons [5]. Indeed Turnier et al. in 2015 described that 28% of the positive results were attributable to rhinovirus/enterovirus, 8.7% due to parainfluenza and the remaining pathogens: respiratory syncytial virus, influenza, adenovirus and human coronavirus (strains 229E, HKU1, NL63, OC43) were each positive less than 5% of the time [6].

## Main text

Following the outbreak of SARS-CoV-2 infection, COVID-19 pandemic is emerging as a global health issue. In this context the scientific community is wondering about a possible correlation between SARS-CoV-2 virus infection and the onset of Kawasaki-like diseases. From studies reported so far, the pediatric population appears to be affected less than adults. In the last period with the increase in number of infections, there have been first reports of KD secondary to SARS-CoV-2 infection [7], some typical forms, other atypical ones. Shelley Riphagen et al. during a 10-day period in mid-April, in the UK, reported a cluster of 8 children with hyperinflammatory shock syndrome. This cluster of cases formed the basis of a national alarm. They suggest that this clinical picture represents a new phenomenon that affects previously asymptomatic children with SARS-CoV-2 infection manifesting itself as a hyperinflammatory multi-organ involvement syndrome similar to a shock syndrome in KD [8]. Kawasaki disease shock syndrome (KDSS) is characterized by cardiovascular shock, associated with resistance to immunoglobulins, coronary anomalies and hyperinflammatory state with possible cardiovascular shock [9]. In studies of adult patients with Covid-19, a subset of patients showed hyperinflammation and multi-organ failure due to excessive release of cytokines caused by an uncontrolled immune response. Similarly, genetically susceptible pediatric patients, after contact with Covid-19, could develop a disease complicated by hyperinflammatory shock. KD, as mentioned above, is known to recognize infectious triggers, most often viruses, and SARS-COV 2, which is, at present, the most common infectious agent in the world, could probably induce the development of epidemic clusters outbreaks by KD. The recorded anomaly could be the significant percentage of severe KD cases: this could be explained by the fact that the typical Covid-19 cytokine storm has a substantial overlap with that of Kawasaki disease, with high levels of IL-1, IL-6 and TNF-alpha, and also the presence of circulating activated macrophages that characterize both the diseases [10].

## Conclusions

It is therefore necessary, in order to avoid diagnostic and therapeutic pitfalls, in the presence of a child with symptoms compatible with KD, to exclude an infection with SARS-CoV-2 virus, even in the presence of an initial negativity of the search for SARS-CoV-2, through nasopharyngeal swab and serological research tests. Of note, epidemiological studies are awaited in order to avert overestimated prevalence of KD correlated somehow with Covid-19 infection. In the meantime several medical societies have issued strict statements with the aim of either accurate diagnosis and appropriate treatment.

## Data Availability

Not applicable.

## Abbreviations

KD: Kawasaki Disease
AHA: American Heart Association

## References

[1] Newburger JW, Takahashi M, Gerber MA (2004). Diagnosis, treatment, and long-term management of Kawasaki disease: a statement for health professionals from the Committee on Rheumatic Fever, Endocarditis, and Kawasaki Disease, Council on Cardiovascular Disease in the Young, American Heart Association [published correction appears in Pediatrics. 2005 Apr;115(4):1118]. Pediatrics.

[2] McCrindle BW, Rowley AH, Newburger JW (2017). Diagnosis, treatment, and long-term management of Kawasaki disease: a scientific statement for health professionals from the American Heart Association [published correction appears in Circulation. 2019 Jul 30;140(5):e181-e184]. Circulation.

[3] Matsubara T, Ichiyama T, Furukawa S (2005). Immunological profile of peripheral blood lymphocytes and monocytes/macrophages in Kawasaki disease. Clin Exp Immunol.

[4] Franco A, Shimizu C, Tremoulet AH, Burns JC (2010). Memory T-cells and characterization of peripheral T-cell clones in acute Kawasaki disease. Autoimmunity.

[5] Nakamura Y (2018). Kawasaki disease: epidemiology and the lessons from it. Int J Rheum Dis.

[6] Turnier JL, Anderson MS, Heizer HR, Jone PN, Glode MP, Dominguez SR (2015). Concurrent respiratory viruses and Kawasaki disease. Pediatrics.

[7] Verdoni L, Mazza A, Gervasoni A, et al. An outbreak of severe Kawasaki-like disease at the Italian epicentre of the SARS-CoV-2 epidemic: an observational cohort study [published online ahead of print, 2020 May 13]. Lancet. 2020. 10.1016/S0140-6736(20)31103-X. 10.1016/S0140-6736(20)31103-X PMC7220177 32410760

[8] Riphagen S, Gomez X, Gonzalez-Martinez C, Wilkinson N, Theocharis P. Hyperinflammatory shock in children during COVID-19 pandemic [published online ahead of print, 2020 May 7]. Lancet. 2020. 10.1016/S0140-6736(20)31094-1. 10.1016/S0140-6736(20)31094-1 PMC7204765 32386565

[9] Gamez-Gonzalez LB, Moribe-Quintero I, Cisneros-Castolo M (2018). Kawasaki disease shock syndrome: unique and severe subtype of Kawasaki disease. Pediatr Int.

[10] Henderson LA, Canna SW, Schulert GS, et al. On the alert for cytokine storm: immunopathology in COVID-19 [published online ahead of print, 2020 Apr 15]. Arthritis Rheumatol. 2020. 10.1002/art.41285. 10.1002/art.41285 PMC7262347 32293098

